# Iodine Availability through Iodized Salt in Portugal: 2010–2021 Sales Evolution and Distribution

**DOI:** 10.3390/nu15061324

**Published:** 2023-03-08

**Authors:** Sarai Isabel Machado, Maria Lopes Pereira, Susana Roque, Maria José Costeira, Adriano A. Bordalo, André Miranda, Patrício Costa, Nuno Borges, Joana Almeida Palha

**Affiliations:** 1 Life and Health Sciences Research Institute (ICVS), School of Medicine, University of Minho, 4710-057 Braga, Portugal; 2 ICVS/3B’s-PT Government Associate Laboratory, 4710-057 Braga, Portugal; 3 Clinical Academic Center-Braga (2CA-B), 4710-243 Braga, Portugal; 4 Hospital da Senhora da Oliveira-Guimarães, 4835-044 Guimarães, Portugal; 5 Laboratory of Hydrobiology and Ecology, Institute of Biomedical Sciences (ICBAS-UP) & CIIMAR, University of Porto, 4050-313 Porto, Portugal; 6 Faculty of Nutrition and Food Sciences, University of Porto, 4150-180 Porto, Portugal

**Keywords:** iodine deficiency, iodine intake, salt iodization, public health

## Abstract

Salt iodization programs are considered the most cost-effective measures to ensure adequate iodine intake in iodine-deficient populations. Portuguese women of childbearing age and pregnant women were reported to be iodine-deficient, which led the health authorities, in 2013, to issue a recommendation for iodine supplementation during preconception, pregnancy and lactation. In the same year, iodized salt became mandatory in school canteens. Of note, no regulation or specific programs targeting the general population, or the impact of iodized salt availability in retailers, are known. The present study analyzed iodized salt supermarket sales from 2010 to 2021 from a major retailer, identifying the proportion of iodized salt in total salt sales and its distribution in mainland Portugal. Data on iodine content were collected through the nutritional label information. Of a total of 33 salt products identified, 3 were iodized (9%). From 2010 to 2021, the weighted sales of iodized salt presented a growing tendency, reaching the maximum of 10.9% of total sales (coarse plus fine salt) in 2021. Iodized salt reached a maximum of 11.6% of total coarse salt in 2021, a maximum of 2.4% of the total fine salt in 2018. The overall sales of iodized salt and their contribution to iodine intake are extremely low, prompting additional studies to understand the consumer’s choice and awareness of the benefits of iodized salt.

## 1. Introduction

Iodine is an essential micronutrient, crucial for the synthesis of thyroid hormones, which are hormones that regulate various metabolic processes throughout life, including the proper development of the central nervous system [1]. Iodine deficiency represents, after starvation, the most relevant and preventable single cause of mental retardation, not allowing the complete development of the individual’s intellectual potential, and having deleterious effects even when mild or moderate [2,3,4]. The recognition of the broad consequences, and potential impact on public health, highlights the need to develop strategies to prevent the spectrum of impairments caused by iodine deficiency [5,6].

### 1.1. Iodine Intake Recommendations, Dietary Sources, and Deficiency Prevention Strategies

The United Nations Children’s Fund (UNICEF), the Iodine Global Network (IGN) (previously the International Council for the Control of Iodine Deficiency Disorders (ICCIDD)), and the World Health Organization (WHO) all recommend an iodine daily intake of 90 μg/day for preschool children, 120 μg/day for school children, 150 μg/day for adolescents and adults, and 250 μg/day during pregnancy and lactation. The European Food Safety Administration (EFSA) has proposed 200 μg/day for pregnant women, assuming a sufficient intake prior to pregnancy [7,8,9,10].

Iodine nutritional requirements can be achieved through food given that it is a natural source. However, the iodine content of foods is highly dependent on the iodine content of the soil and water [11]. Milk, fish, and eggs are good sources of this micronutrient, while fruit, vegetables and bread also provide iodine, even if in smaller amounts [12]. Nonetheless, because the iodine content in food differs between countries, it is important to address and personalize intake recommendations. Besides iodine already naturally present in foods, fortification, especially through iodized salt, has become an important strategy in many countries to prevent iodine deficiency [8].

On this, salt iodization represents the most widely used and cost-effective measure to prevent iodine deficiency [8,13]. Not only does fortification through salt reach all population groups (salt intake is relatively stable), but also salt iodization is a process that is practical to monitor at production and render available through retail to reach household contexts [14]. Furthermore, the ability to adjust the iodine content in salt makes it compatible with sodium intake reduction programs to prevent cardiovascular diseases [15].

The WHO recommends universal salt iodization from 20 to 40 mg/kg, including salt used in food production, and that this measure should cover >90% of households [8,13]. Nevertheless, to achieve the ultimate goal of controlling and eliminating iodine deficiency disorders, iodized salt has to reach the entire population, particularly the more vulnerable groups, such as children and pregnant women [16].

Supplementation may also be an important strategy to prevent iodine deficiency, particularly in countries with less than 20% of households covered by salt iodization programs, and specifically targeting vulnerable population groups who are at higher risk of iodine deficiency [9].

### 1.2. Global Iodine Status

The iodine status of a country can be assessed by the median urinary iodine concentration of school-age children, classifying the overall population risk (or not) of deficiency when compared to the categories set by the WHO [8]. Nonetheless, it is important to take into consideration the limitations of using this method for evaluation, as it may not be appropriate considering that the intake of iodine from dietary sources varies according to the age group [17]. In addition, specific groups, such as pregnant women, have higher dietary iodine requirements and their iodine status should not be extrapolated from data on school-age children [17].

In the last decade, great progress was made, particularly due to the successful implementation of salt iodization programs. Recent data identified 126 countries with mandatory salt iodization and 21 with voluntary programs [18]. The actions taken have led to great progress in preventing iodine deficiency. Countries presenting iodine deficiency now total 21 (and thus classified as an “outstanding and under-recognized public health achievement” [19]), compared to the 118 presenting adequacy [20]. Notwithstanding, to sustain results, preventing deficiency and excess, it is important to continue monitoring, promoting, and adjusting these programs.

### 1.3. Iodine Status and Iodine Deficiency Prevention Strategies in Portugal

In Portugal, both women of childbearing age and pregnant women are considered iodine-deficient [3,21,22]. This led the national health authorities to issue, in 2013, a recommendation for iodine supplementation (150 to 200 μg/day) in women during preconception, pregnancy, and lactation [23,24]. In the same year, a specific program enforced that iodized salt should be implemented in school canteens [25]. For the general population, salt iodization remains voluntary and without monitoring measures.

Of note, the most recent data suggest that despite women’s adherence to the iodine supplementation recommendation, pregnant women are still iodine-insufficient [26,27]. As for children, a recent study points to iodine sufficiency (median urinary iodine concentration of 129 μg/L), even though 32% of children were still found with urinary iodine levels considered insufficient.

The adherence of school canteens to the use of iodized salt is of concern. A study in Northern Portugal evaluated salt samples of 83 canteens; of these, none were using iodized salt, demonstrating that a regulation framework and surveillance is lacking, and that the efficacy of this policy may not be as expected. Data from the same study indicate that only 8% of the parents surveyed reported the usage of iodized salt; of these, for only 20% were data corroborated by the analysis of the household salt sample provided [28].

Given that iodine intake from salt may be a considerable source of iodine in the diet, the present study analyzed iodized salt availability and sales from supermarkets, in Portugal, through the information retrieved from a major retailer, in the period 2010–2021.

## 2. Materials and Methods

Data were provided by Jerónimo Martins, SGPS, SA, and refer to household salt sold in the supermarket Pingo Doce, from January 2010 to December 2021, in mainland Portugal. Pingo Doce is a leading chain in the supermarket segment directed to households in Portugal, and customers are representative of the Portuguese population.

Pingo Doce accounted for 25% of the total national salt market share in the last five years, 24.3% in 2016, 23.0% in 2015, 23.1% in 2014, 21.8% in 2013, and 22.5% in 2012, as reported by the Nielsen consulting company. We estimated the mean values of the market share in 2012-2021 (24%) to input the value for years 2011 and 2010.

The information retrieved includes product descriptions and classification as coarse or fine salt, categorized by monthly sales [(kg)]. Sales data were aggregated by district and not linked to individuals.

A total of 53 salt products were identified. In the analysis, products classified as salt blends, samphire, sodium bicarbonate, and liquid salt were excluded, given that they only represented 0.02% of the total sales from 2010 to 2021. A total of 33 salt products were thus considered in the analysis.

The iodine content by 100 g of product was collected through the nutritional label information. From the 3 iodized salt products, the coarse salts had an iodine content of 23.8 mg/kg and of 23.0 mg/kg, and the fine salt of 23.0 mg/kg.

Estimates of household salt per capita (g/day) were calculated by the amount of salt sold in the year converted into grams, divided by 365 (number of days of the year) and the mean resident population older than four years in each year (mainland Portugal), considering the market share of each year.

Estimates of iodine per capita (μg/day) were calculated by the amount of iodine in the salt sold in the year converted into micrograms, divided by 365 (number of days of the year) and the mean resident population older than four years in each year (mainland Portugal), considering the market share of each year.

Data from the annual average population resident from 2010 to 2021 were exported from Pordata, INE (National Institute of Statistics), subtracting the population resident with ages equal to or below 4 years of age (this is the lower age interval and includes children that may be breastfeeding and as such receiving iodine through the mother).

A descriptive analysis of sales was performed using Microsoft^®^ Excel for Mac, Version 16.61.1. Statistical analysis was performed using IBM SPSS Statistics, 26.0 for Mac, to verify if the mean slope of the estimates of iodine per capita (μg/day) was significantly greater than zero, by a one-tailed one-sample *t*-test.

## 3. Results

### 3.1. Iodized Salt Sales from 2010 to 2021

As shown in Table 1, a total of 33 salt products were identified, of which 3 were iodized (9%), 2 in the form of coarse salt, and 1 as fine salt. Most sales were in the format of coarse salt (94.8%). Iodized salt represented 4.0% of the total coarse salt and 0.1% of the total fine salt.

Two of the three iodized salt products were already sold in 2010, and the third, corresponding to the retailer’s own brand of coarse iodized salt, was introduced in the market in 2018.

The percentage of iodized and non-iodized salt in total salt sales is characterized in Table 2. It shows that total non-iodized salt sales decreased, while the percentage of iodized salt correspondingly steadily increased.

### 3.2. Iodized Salt Sales by District

Only five districts reported sales of iodized salt from 2010 to 2012, a number that greatly increased in 2013, to 16, reaching all 18 mainland Portuguese districts in 2016.

**Figure 1:**
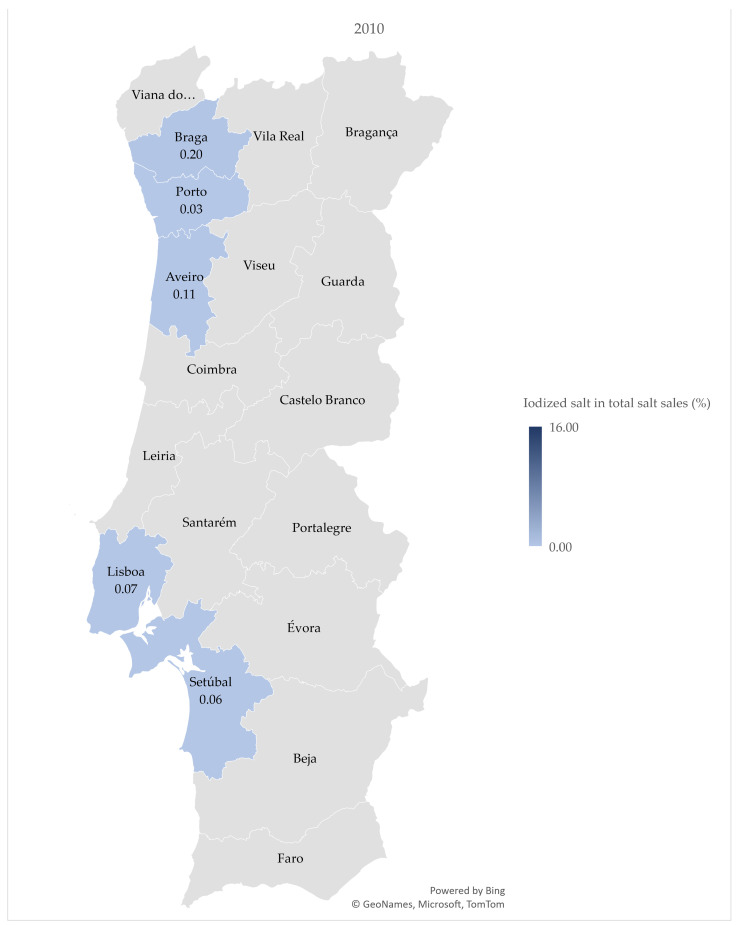
Percentage of iodized salt in total salt sales by district in 2010.

**Figure 2:**
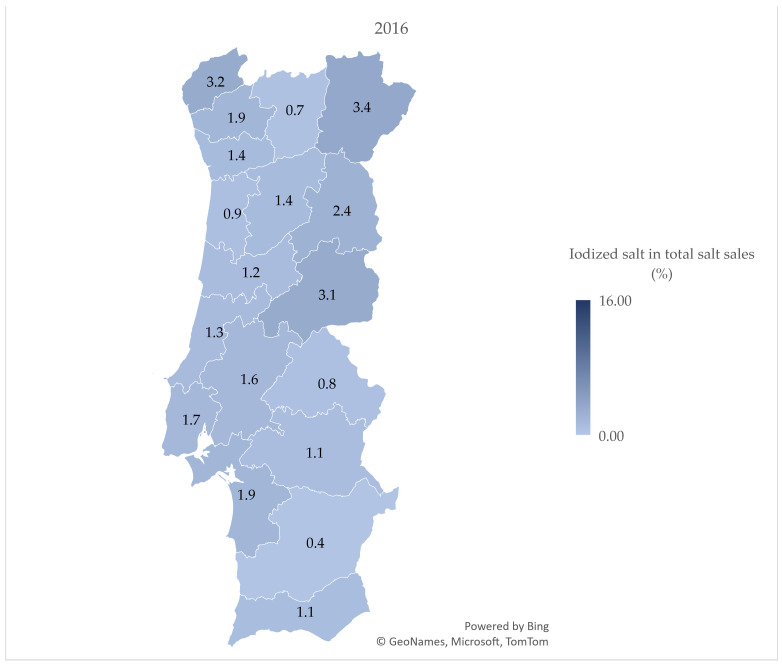
Percentage of iodized salt in total salt sales by district in 2016.

**Figure 3:**
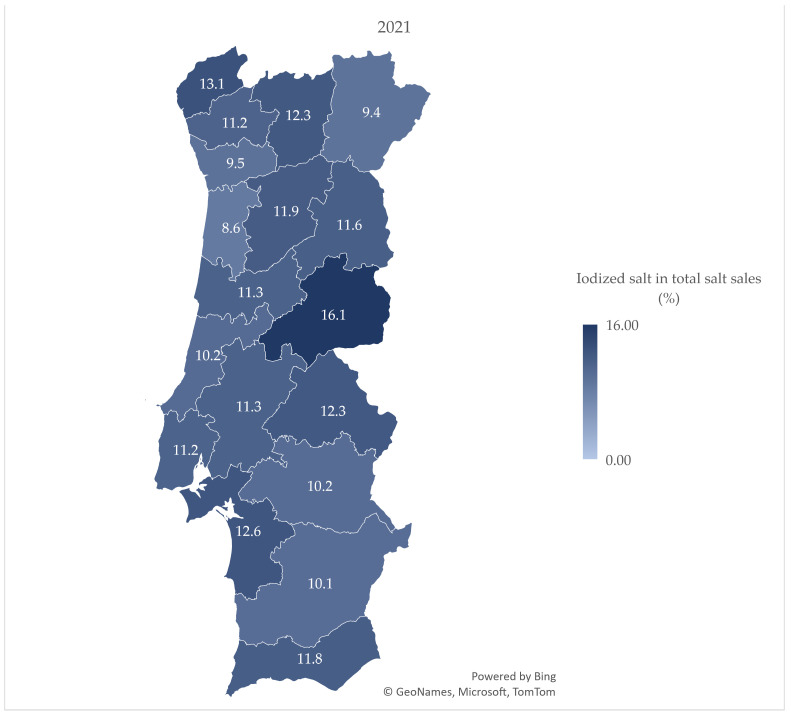
Percentage of iodized salt in total salt sales by district in 2021.

Figure 1, Figure 2 and Figure 3 demonstrate the variation in the percentage of iodized salt in total salt sales in the 18 mainland Portuguese districts, corresponding to the years of 2010, 2016, and 2021, respectively. Figure 3 shows the percentage of iodized salt sold in 2021, with a value range between 8.6 (Aveiro) and 16.1 (Castelo Branco).

### 3.3. Estimates of Household Availability of Salt and Iodine per Capita, from 2010 to 2021

Next, we calculated an estimation of total salt and iodine intake per capita, as shown in Table 3. Total salt intake per capita slightly increased until 2016 and presented a steady, slow decrease thereafter. Of note, the estimated iodine intake from salt increased considerably. Analyzing the mean slope of the 18 mainland Portuguese districts (t (17) = 13.8, *p* < 0.001, d = 0.7), each year, there was an increase of 2.4 μg/day of iodine per capita.

## 4. Discussion

### 4.1. Iodized Salt Sales from 2010 to 2021

The present study provides information about estimated changes in salt and iodine intake per capita, through supermarket sales and the market share of iodized salt, in the last decade, in mainland Portugal, revealing increased consumption, mostly through coarse salt. In fact, the percentage of iodized fine salt in the total fine salt sales remained low and fine salt per se is not a great contributor to salt sales and intake, as most household salt is coarse. The use of fine salt is commonly restricted to the seasoning of salads and other already cooked meals, to increase the salty flavor.

The percentages of iodized salt sales identified in 2015 and 2016 are lower compared to the reported iodized salt usage in households, in the same years, by the parents of school-age children that participated in a study in Northern Portugal (0.93% and 1.55% vs. 8%). However, the results are in accordance with what was corroborated by household salt samples (0.93% and 1.55% vs. 1.6%). The disparity is also seen in the reports of the National Food and Physical Activity Survey (IAN-AF) in 2015, as the unpublished results indicated 15% [29]. It is important to consider that self-reports are influenced by knowledge about what iodized salt is and by social desirability.

For 2019, the results indicated an iodized salt sales percentage of 9.3%, considerably lower than what is reported in the Azores islands, also in 2019, where almost half of the households confirmed the usage of iodized salt (48.3%) [28,29,30]. In fact, in the Azores, the local government implemented several strategies, alongside mandatory usage in meals prepared by the Regional Directorate of Education, to promote the consumption of iodized salt—for instance, mandatory usage in the catering of the Regional Health Service, media advertising, and the distribution of educational material, promoting literacy regarding iodine deficiency’s consequences, highlighting the role of iodized salt and iodine-rich food intake [30]. Moreover, the comparison of urinary iodine concentrations in school children, before and after these measures, corroborates the effectiveness of the program [28]. It should be noted that, however, even with the investment in information dissemination regarding iodized salt, a recent study in one of the Azores islands revealed, in 2020, that 32% of approached individuals did not know about the existence of iodized salt and/or the benefits of iodized salt usage [31].

Nonetheless, the percentages presented a contrast with the iodized salt usage percentage of the global population estimated by UNICEF (88%), as well as the WHO recommendation that these programs should aim to cover more than 90% of households [8]. Of note, the WHO recommends countries in which less than 20% of the household have access to iodized salt to assess the current situation of its salt iodization program, if any, to identify problems and action plans. In such cases, supplementation should be given as a supplement or complementary food fortified with iodine until the strengthening of the iodization program [9]. This seems to be the case of Portugal.

In our study, the observed growth in iodized salt sales may result from the increased availability of iodized salt products, the introduction of new iodized salt products with competitive prices, or/and an informed decision given the higher awareness of the benefits of iodized salt, which could be mediated by information given by health professionals or even media coverage and advertising. It is also important to note that iodized salt is on average 73% more expensive than the equivalent non-iodized salt, as this may be a factor influencing choices. Nevertheless, iodized salt costs less than other products, such as flower of salt (which costs on average 11 times more per kg) or Himalayan salt (which costs on average 4 times more per kg).

Ongoing research within the IodineMinho study is addressing customers’ motivation, intention, and awareness regarding the choice to purchase iodized salt, and the intake of iodine from food through food diaries and food frequency questionnaires [32]. Iodine intake data, through dietary assessment, complement the evaluation of iodine status, and allow a better perception of the key points that determine adequacy and should be targeted by public health strategies [17].

### 4.2. Iodized Salt Sales by District

The % of iodized salt does not vary greatly across districts. Although we do not have data on the distribution of supermarket stores across the territory, the accessibility to the locations could differentiate the costumer characteristics in each district, as well as the market share by district. Nevertheless, we expect that the percentage of iodized salt in the total salt sales may not vary considerably.

Castelo Branco presented the higher proportion of salt sales (together with the Minho region and Setúbal); interestingly, Castelo Branco was the only district in which legislation in 1969 made mandatory the use of iodized salt due to an endemic goiter report; still, iodized salt sales started only in 2013 [33]. In addition, Algarve does not present a % above average, even though the majority of the household salt in Portugal is produced in this region [34].

### 4.3. Estimates of Household Availability of Salt and Iodine per Capita, from 2010 to 2021

Analyzing overall per capita daily salt intake estimates, we observed an estimated value of 6.8 g in 2021. The last available data from the Portuguese population indicate a daily salt intake per capita of 10.7 g [35]. Both estimates are well above those recommended by the WHO for salt consumption (<5 g) [35,36]. It is important to note that the data from the present study only represent household supermarket sales, not incorporating salt from other food sources, such as processed foods, that are relevant contributors to salt intake. For instance, in Portugal, bread accounts for one sixth of the total salt intake [37,38]. Total salt sales started to decrease in 2016, by the time that national strategies to reduce salt intake initiated [39]. The agreements with producers and restaurants to implement monitoring practices of salt content were completed in 2016 [39].

Excessive salt intake is a major cause of non-communicable diseases, such as cardiovascular diseases and hypertension. It is, therefore, relevant to balance the implementation and reinforcement of the consumption of iodized salt and, simultaneously, potentiate a reduction in total salt intake [40,41]. The WHO reinforces that both measures are compatible and demand integrated actions, referred to as “double duty actions” [16]. Furthermore, a balance may only be achieved through a structural program, highlighting the need for continuous monitoring, adjusting the amount of iodine in fortification as a response to decreases in population salt intake [15]. In fact, the need to combine efforts, not only implementing strategies to reduce salt but also to ensure iodine availability through salt, was already identified by the WHO Action Network on Salt Reduction in the Population in the European Region in 2016 [39].

When considering the contribution of household iodized salt to daily iodine intake, we observed an estimated maximum of 17.6 μg/day, achieved in 2021. This value is far from what is the UNICEF/ICCIDD/WHO recommended daily intake, representing only 14.7% of the needs for adolescents and adults (150 μg/day) and 8.8% during pregnancy and lactation (250 μg/day) [8]. Results from the ATLAS study in European lactating women also indicated that iodine intake is not compliant with the recommendations [42].

The iodine content present in Portuguese foods was updated in 2018, and showed seafood and dairy as the products having higher levels of iodine [12]. For Portuguese pregnant women, dairy products, particularly milk, are important iodine sources [43]. However, consumption may not be sufficient to achieve the recommended amounts [43]. Regarding fish consumption, studies show no association with iodine status, as intake appears to be low and the variability of iodine content of fish, particularly aquaculture fish, may also explain this lack of contribution [3,43]. Recent data analyzing meal compositions in school-aged children indicate that the preparation of at least one meal with iodized salt is necessary in order to ensure the adequate iodine intake recommendations [44]. Dietary patterns with more strict restrictions and without monitoring may increment the risk of inadequate iodine intake and insufficient iodine status [45]. As adherence to plant-based dietary patterns becomes increasingly popular, the implications regarding the achievement of adequate iodine intake should be considered and incorporated into strategies of communication at a public health level [17].

It would also be important to consider the utilization of iodized salt in restaurants and by the industry as, to our knowledge, there are no data in Portugal.

The present study suggests that the iodine intake contribution from household iodized salt usage is lower than what is reported in other populations, where specific recommendations have been issued concerning iodized salt, namely in Germany (52.2 μg/day) and Italy (54.9 μg/day) [46,47,48]. Interestingly, even countries with no specific iodized salt policies (such as the United Kingdom) report higher percentages of iodized salt sales (21.5%) than those here reported [49].

Across Europe, only 40% of countries have mandatory salt iodization policies [17]. In the platform of global fortification data exchange, it is recognized that salt represents an important fortification opportunity for Portugal to achieve the necessary health targets. Nevertheless, there are no fortification compliance data, leaving the suggestion that a greater impact may be achieved through mandatory polices [18]. It is, nonetheless, important to note that other approaches can be taken [17]. Such is the case of the Netherlands, where it was possible to ensure the fortification of almost all bread with iodized salt, representing an important source of iodine [50].

A final note on the salt iodine content. Nutritional information of the natural iodine content of salt is not mandatory. The available data indicate that the non-fortified salt samples, even when considering marine salt, have minimal levels of iodine and do not achieve the values indicated by the WHO [34]. As for fortified salt, the three iodized salts presented label values within the 15–40 mg/kg recommendation by the WHO at a household level, in accordance with what has been previously shown [8], but below the Portuguese recommendation of fortifying salt with KI from 25 mg/kg to 35 mg/kg, representing 19 mg/kg to 26.6 mg/kg of iodine [51]. In fact, these values are significantly lower than what is recommended by the United States Food and Drug Administration (46 to 76 mg/kg) or what is practiced in Spain (60 mg/kg) [52,53]. Of relevance, the amount must be regulated in each country, considering the salt intake and the type of policy taken (whether addressing salt for households or for the food industry), and the iodine status of the population [15].

In summary, estimates of iodine intake per capita suggest that iodized salt from household supermarket sales is still a minor contributor to iodine intake in the Portuguese population, as iodized salt sales are still low.

The present study calls the attention of the health authorities to the opportunity to develop campaigns and programs and/or issue specific recommendations on the use of iodine fortified salt as a relevant strategy to ensure adequate iodine intake and a sufficient iodine status of the population, including groups at higher risk.

### 4.4. Limitations

This study presents several limitations. Data were made available for this study from a single retailer that represents approximately 25% of the salt market share in a specific period, so direct extrapolations may not reflect the precise iodine sales behavior for the whole population. Furthermore, the availability of iodized salt may also depend on marketing strategies, for which we have no information.

Total sales do not necessarily correspond to individual consumption, since salt acquired in the supermarket can be used for various culinary processes that may not lead to intake (e.g., preservation, culinary processes), rather than direct consumption. In addition, we have no information on the salts used in the food industry and on how much of these may contribute to the daily iodine intake; it should, however, be minimal.

Regulation of nutritional labeling is not mandatory regarding the content of iodine naturally presented in foods. Moreover, the iodine content of salt products could be different over the years. We assumed the values indicated in the nutritional label information. In addition, we did not account for the iodine content in non-iodized salt, which, in any case, is residual.

The time of storage and storage conditions influence the amount of iodine available for consumption. The present study did not control for shelf-time or conditions; therefore, the results obtained are likely to represent an overestimation of the actual iodine obtained through household iodized salt.

## Figures and Tables

**Table 1:** Salt sales from 2010 to 2021.

Salt Type	No. of Salt Products	Sales (kg)	Sales (%)
**Iodized salt**	**3**	**2,815,956**	**4.1**
Coarse	2	2,771,297	4.0
Fine	1	44,659	0.1
**Non-iodized salt**	**30**	**66,299,501**	**95.9**
Coarse	20	62,767,079	90.8
Fine	10	3,532,421	5.1
**Total**	**33**	**69,133,401**	**100**

**Table 2:** Percentage of salt sales in total salt sales (%) from 2010 to 2021.

	Percentage of Salt Sales in Total Salt Sales (%)					
Year	Total Non-Iodized Salt	Coarse Non-Iodized Salt	Fine Non-Iodized Salt	Total Iodized Salt	Coarse Iodized Salt	Fine Iodized Salt
2010	99.95	94.62	5.33	0.05	0.05	0.01
2011	99.94	94.65	5.29	0.06	0.05	0.01
2012	99.94	94.88	5.06	0.06	0.05	0.01
2013	99.74	94.89	4.85	0.26	0.24	0.02
2014	99.39	94.51	4.89	0.61	0.56	0.04
2015	99.07	94.23	4.85	0.93	0.87	0.05
2016	98.45	93.65	4.80	1.55	1.48	0.07
2017	95.41	90.72	4.68	4.59	4.52	0.08
2018	91.03	86.57	4.46	8.97	8.86	0.11
2019	90.70	85.65	5.05	9.30	9.18	0.12
2020	89.65	83.73	5.92	10.35	10.22	0.13
2021	89.08	82.72	6.36	10.92	10.80	0.12

**Table 3:** Estimates of household availability of salt and iodine per capita, from 2010 to 2021.

Year	Population Older than Four Years of Age	Estimates of Salt per Capita (g/day)	Estimates of Iodine per Capita (μg/day)
2010	9,579,729	6.0	0.1
2011	9,576,046	6.2	0.1
2012	9,543,700	7.2	0.1
2013	9,499,685	7.6	0.5
2014	9,460,800	7.3	1.0
2015	9,433,777	7.7	1.6
2016	9,413,499	7.2	2.6
2017	9,395,108	7.0	7.4
2018	9,379,021	6.8	14.5
2019	9,376,734	6.9	15.2
2020	9,437,325	6.9	16.9
2021	9,484,013	6.8	17.6

## Data Availability

Third-party data. Restrictions apply to the availability of these data. Data were obtained from Pingo Doce and are available from the authors upon review and with permission from Jerónimo Martins.
